# Supplementary material for: Harnessing the Antibacterial Properties of Fluoridated Chitosan Polymers against Oral Biofilms
Source: Pharmaceutics. 2022 Feb 23;14(3):488. doi: 10.3390/pharmaceutics14030488 (PMC8951426; doi:10.3390/pharmaceutics14030488)
Supplement: Supplementary file 1 [file pharmaceutics-14-00488-s001.zip › pharmaceutics-1594441-supplementary.pdf]

pharmaceutics

Supplementary Materials: Title

Dien Puji Rahayu, Roger Draheim, Aikaterini Lalatsa and Marta Roldo

S1. Synthesis of *N*-2(2,6-diaminohexanamide)-chitosan (CS3H Lys)

To a solution of Fmoc-protected lysine (1.54 g, 2.6 mmol, 1 eq) in *N*-methyl-2-pyrrolidone (NMP, 20 mL), stirred at room temperature for 1h to allow complete dissolution, the following were added: EDC (0.85 g, 4.42 mmol, 1.7 eq) in 4 ml of NMP, and NHS (0.15 g, 1.3 mmol, 0.5 eq) in 1 ml of NMP. The reaction was left to proceed under stirring at room temperature for 3 h and was protected from light. CS3H (1% *w*/*v*, 0.5 g) in NMP (50 mL) was added and the pH was adjusted to > 7.5 using NaOH (1M in NMP). The reaction mixture was stirred at 40 °C for further 24 h. Fmoc was then removed by adding piperidine (20% *v*/*v*, 6 ml) and stirring at 40°C for 20 min. The product was filtered with Whatman No. 1 filter paper, and the deprotection process was repeated four times for the filtrate. The product was collected and dialysed (MWCO: 12-14 kDa) against 5 L of distilled water with 6 changes over 24 h. The dialysate was lyophilised, and a white product was obtained.

S1. Determination of fluoride

In the potentiometric measurement for fluoride, the linearity was verified for a wide concentration range from 2 to 1000 µg/mL of fluoride. The result showed the dependence of the potential (mV) versus log concentration (Figure S1). The linear correlation coefficient (r^2) was 1, and the slope had a practical value of −58.755 mV (Nerst equation theoretical value −59.16 mV), indicating an ideal range at 25°C for the determination of fluoride ions.

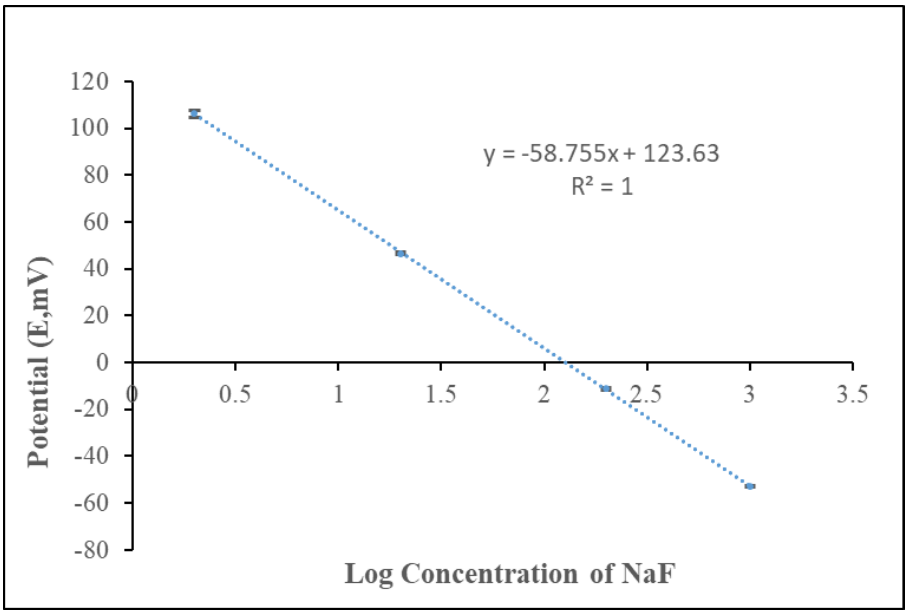

Figure S1. Calibration curve of fluoride ion determination.

In Table S1 is an example of how to calculate the concentration of fluoride ions in the samples, each sample is tested after addition of increasing volumes of standard solution, and the data obtained are used to calculate the$10^{E/S}\cdot(V_0 + V_{std})$ value, these values are then plotted as shown in Figure S2.

Table S1. The calculation for fluoride determination in the unknown solution (data were shown as triplicate experiments).

**Standard added**	**C_{std}**	**V_{std}**	**$C_{std}.V_{std}$**	**E (mV)**	**S**	**E/S**	**$10^{E/S}$**	**V_0+V_{std}**	**$10^{E/S}\cdot(V_0+V_{std})$**
				−14.2		0.241682	1.744543		87.227126
N/A	500	0	0	−14.4	−58.755	0.245086	1.75827	50	87.9134916
				−14.8		0.251893	1.786049		89.30246776
				−16.0		0.272317	1.872049		95.47450726
+ 1 mL	500	1	500	−16.0	−58.755	0.272317	1.872049	51	95.47450726
				−16.2		0.275721	1.88678		96.22576918
				−17.6		0.299549	1.993191		103.6459456
+ 1 mL	500	2	1000	−17.8	−58.755	0.302953	2.008875	52	104.4615063
				−18.1		0.308059	2.032633		105.6968957
				−19.1		0.325079	2.113872		112.0352239
+ 1 mL	500	3	1500	−19.3	−58.755	0.328483	2.130506	53	112.9167974
				−19.5		0.331887	2.14727		113.8053078
				−20.4		0.347204	2.224357		120.1152786
+ 1 mL	500	4	2000	−20.3	−58.755	0.345503	2.215657	54	119.6454728
				−21.0		0.357416	2.27728		122.9731081

Where C_{std} = Concentration of standard; V_{std} = Volume of standard; E = measured potential; S = standard curve slope

To calculate the fluoride concentration of the unknown sample equation (1). S1 is used.

$$C_{unknown} = \frac{C_0 V_0}{V_{unknown}} \quad (1)$$

Where C_0V_0 can be obtained from the intercept with the y axis of the lines in figure S2, so for the triplicates the values would be:

A. y = 0.0165x + 87.232	B. y = 0.0162x + 87.901	C. y = 0.017x + 88.617
$C_0V_0 = \frac{87.232}{0.0165} = 5286.79$	$C_0V_0 = \frac{87.901}{0.0162} = 5425.99$	$C_0V_0 = \frac{88.617}{0.017} = 5212.77$
$C_{unknown} = \frac{5286.8}{50} = 105.74\ \mu g/mL$	$C_{unknown} = \frac{5425.99}{50} = 108.52\ \mu g/mL$	$C_{unknown} = \frac{5212.77}{50} = 104.26\ \mu g/mL$

Therefore, the fluoride concentration in the sample is 106.17 µg/mL ± 2.17.

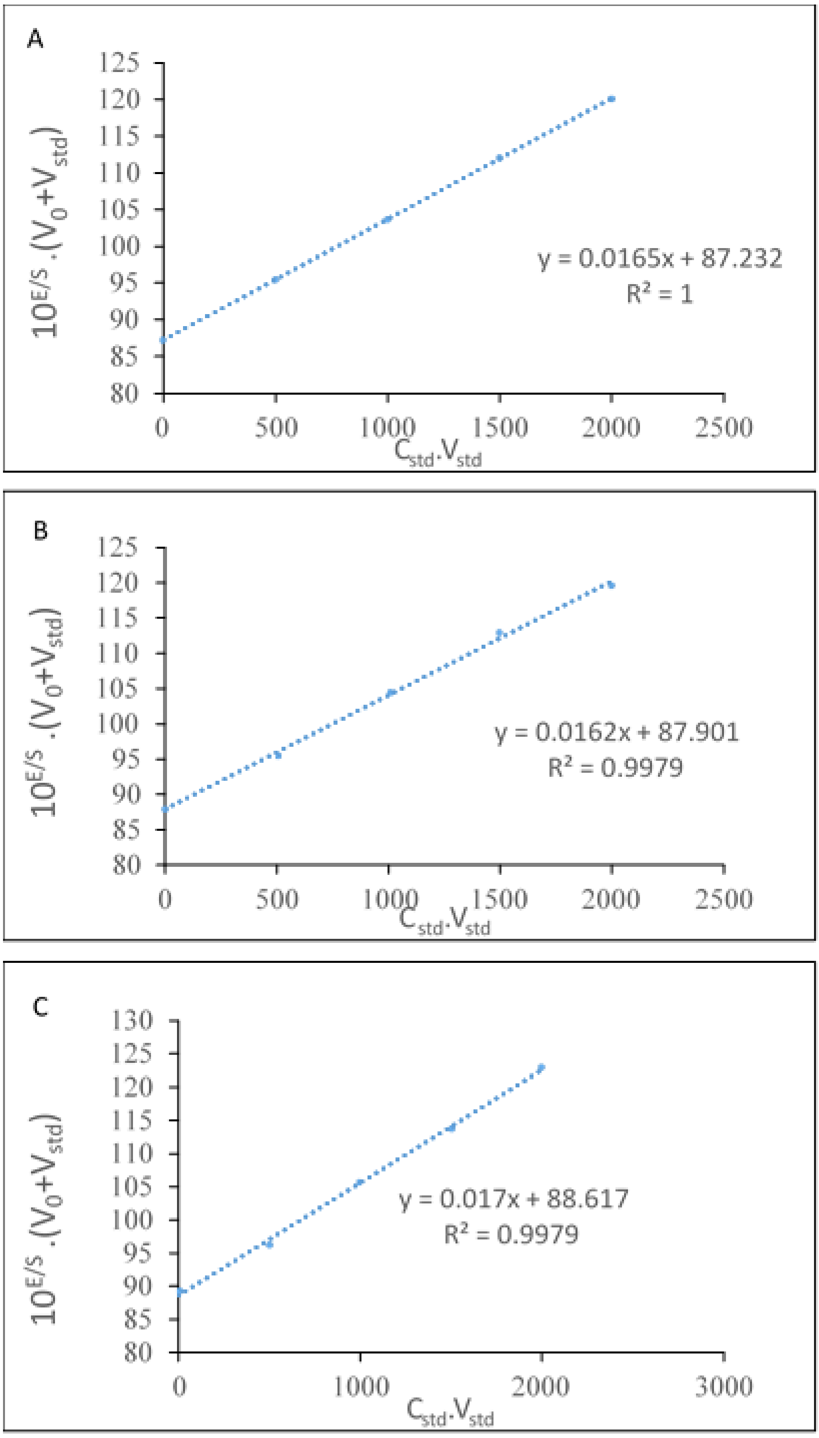

Figure S2. Fluoride ion concentration in the unknown solution.

Evidence of the loading of fluoride ions onto the polymer was also provided by EDX coupled SEM imaging. Samples were sputter coated with Au/Pd (Polaron e500, Quoram Technologies, Lewis, UK) and analysed by SEM, coupled with EDS (Silicon Drift Detector (SDD)−X-MaxN, Oxford Instruments, Abingdon, UK).

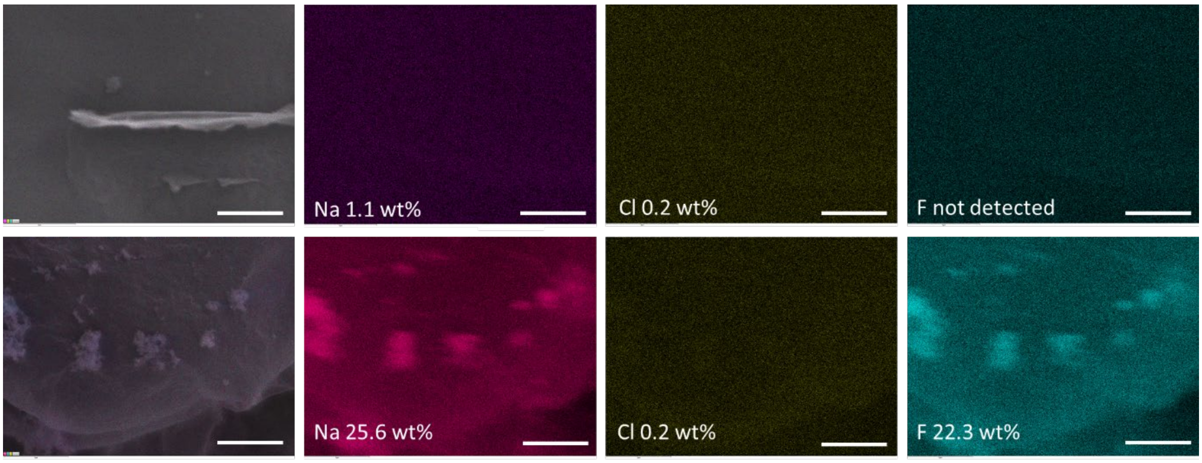

Figure S3. Sample SEM images of CS3H Lys (top) and CS3H Lys F (bottom) and maps showing the distribution of Na, Cl and F with relative % wt at the site of analysis.

S2. UV Measurements to Establish Color Stability of Mouthwash Preparations

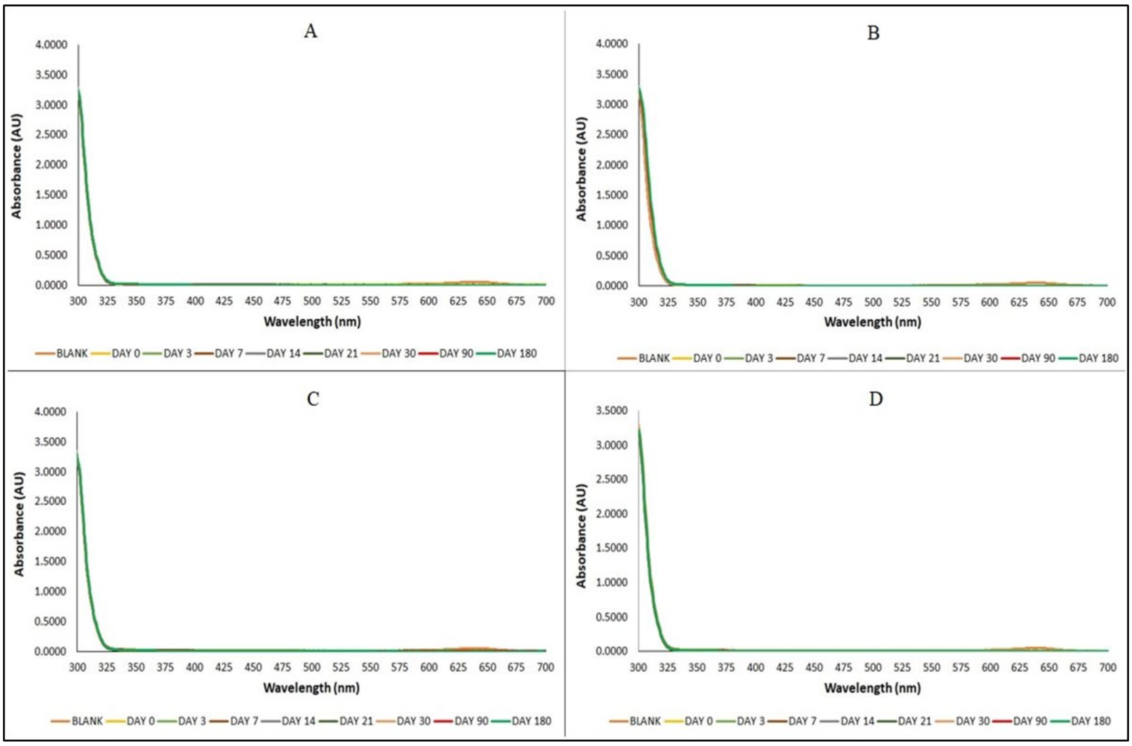

Figure S4. UV absorption spectra of Formula I at room temperature. (**A**) CS, (**B**) CS3H, (**C**) CS3H Lys, and (**D**) CS3H Lys F mouthwash.

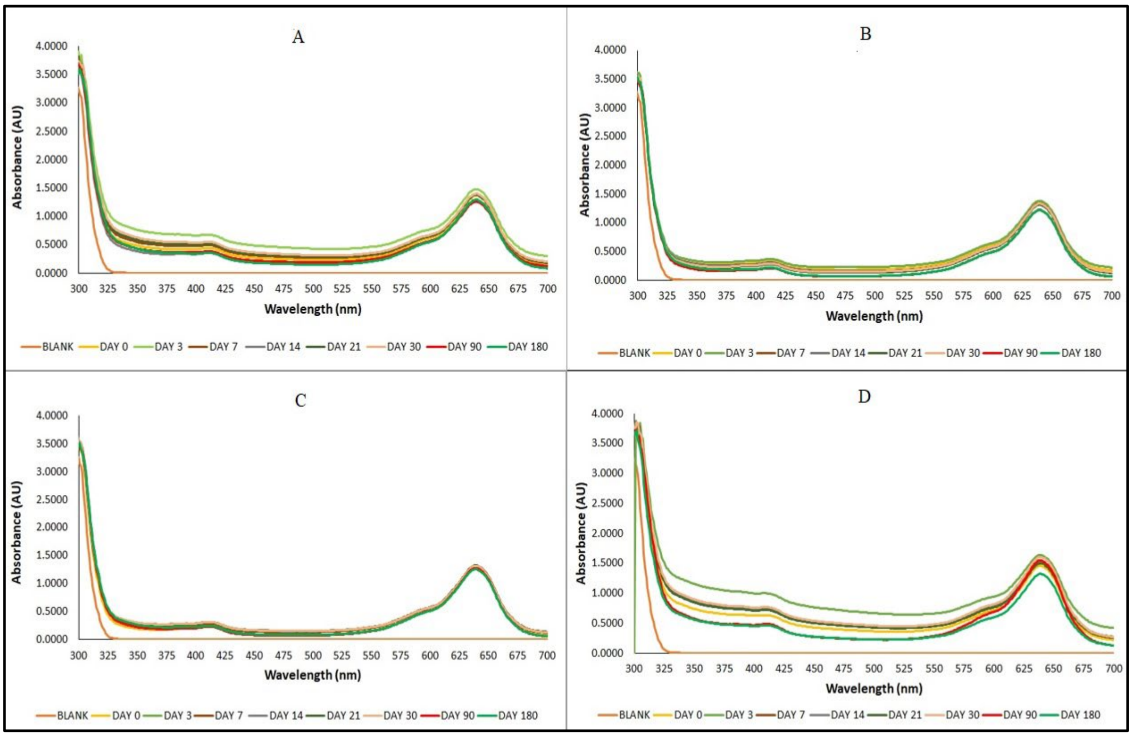

Figure S5. UV absorption spectra of Formula II at room temperature. (**A**) CS, (**B**) CS3H, (**C**) CS3H Lys, and (**D**) CS3H Lys F mouthwash.

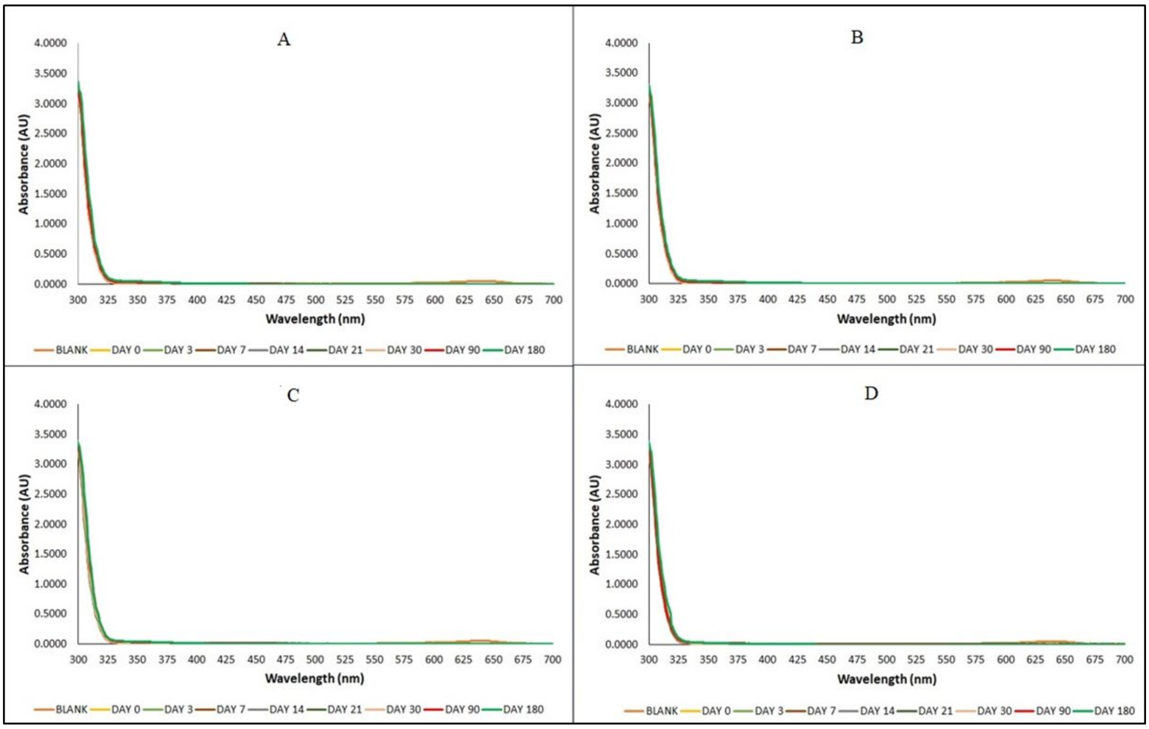

Figure S6. UV absorption spectra of Formula I at 40°C. (**A**) CS, (**B**) CS3H, (**C**) CS3H Lys, (**D**) CS3H Lys F mouthwash.

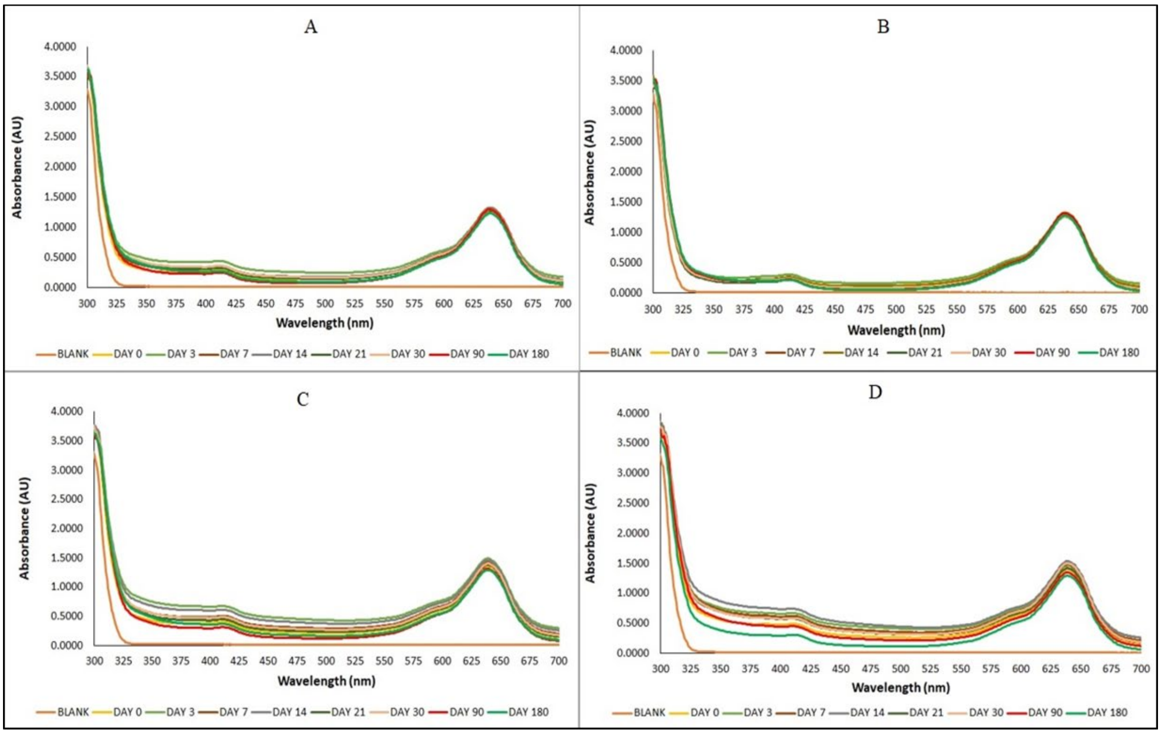

Figure S7. UV absorption spectra of Formula II at 40°C. (**A**) CS, (**B**) CS3H, (**C**) CS3H Lys, (**D**) CS3H Lys F mouthwash .**S3. Determination of pH stability**

Table S2. The effect of temperature on the pH of chitosan mouthwash formulation. Data are expressed as mean ± SD, n = 3.

Polymer	Temp	T_0	T_3	T_7	T_{14}	T_{21}	T_{30}	T_{90}	T_{180}
CS	25°C	5.54 ± 0.02	5.57 ± 0.02	5.55 ± 0.02	5.54 ± 0.02	5.55 ± 0.02	5.56 ± 0.02	5.54 ± 0.02	5.53 ± 0.02
	40°C		5.57 ± 0.02	5.54 ± 0.02	5.52 ± 0.02	5.52 ± 0.03	5.53 ± 0.02	5.52 ± 0.01	5.52 ± 0.03
CS3H	25°C	5.52 ± 0.03	5.54 ± 0.01	5.54 ± 0.01	5.52 ± 0.01	5.53 ± 0.01	5.53 ± 0.01	5.52 ± 0.01	5.52 ± 0.01
	40°C		5.54 ± 0.01	5.52 ± 0.02	5.52 ± 0.01	5.5 ± 0.02	5.5 ± 0.01	5.52 ± 0.01	5.5 ± 0.01
CS3H Lys	25°C	5.52 ± 0.01	5.55 ± 0.01	5.54 ± 0.01	5.52 ± 0.03	5.52 ± 0.02	5.52 ± 0.02	5.52 ± 0.02	5.52 ± 0.02
	40°C		5.56 ± 0.01	5.54 ± 0.01	5.51 ± 0.02	5.48 ± 0.03	5.48 ± 0.01	5.48 ± 0.03	5.48 ± 0.02
CS3H Lys F	25°C	5.52 ± 0.02	5.54 ± 0.05	5.54 ± 0.03	5.53 ± 0.04	5.54 ± 0.03	5.54 ± 0.06	5.54 ± 0.04	5.53 ± 0.05
	40°C		5.53 ± 0.04	5.54 ± 0.04	5.52 ± 0.05	5.51 ± 0.03	5.5 ± 0.04	5.52 ± 0.06	5.51 ± 0.03

Table S3. The effect of temperature on the pH of control mouthwash formulation. Data are expressed as mean values ± SD, *n* = 3.

Polymer	Temp	T_0	T_3	T_7	T_{14}	T_{21}	T_{30}	T_{90}	T_{180}
**CS**	25°C	5.54 ± 0.01	5.57 ± 0.02	5.59 ± 0.02	5.54 ± 0.02	5.56 ± 0.03	5.57 ± 0.03	5.58 ± 0.02	5.62 ± 0.01
	40°C		5.57 ± 0.02	5.58 ± 0.02	5.56 ± 0.04	5.55 ± 0.03	5.55 ± 0.04	5.59 ± 0.02	5.62 ± 0.02
**CS3H**	25°C	5.57 ± 0.01	5.65 ± 0.01	5.64 ± 0.02	5.64 ± 0.02	5.64 ± 0.01	5.64 ± 0.01	5.65 ± 0.01	5.69 ± 0.04
	40°C		5.44 ± 0.01	5.64 ± 0.01	5.64 ± 0.01	5.66 ± 0.01	5.65 ± 0.01	5.67 ± 0.01	5.68 ± 0.05
**CS3H Lys**	25°C	5.51 ± 0.02	5.57 ± 0.03	5.58 ± 0.03	5.57 ± 0.02	5.57 ± 0.03	5.57 ± 0.03	5.62 ± 0.06	5.63 ± 0.02
	40°C		5.59 ± 0.03	5.60 ± 0.02	5.58 ± 0.03	5.59 ± 0.03	5.58 ± 0.03	5.62 ± 0.05	5.64 ± 0.05
**CS3H Lys F**	25°C	5.52 ± 0.01	5.63 ± 0.02	5.64 ± 0.02	5.64 ± 0.03	5.65 ± 0.03	5.65 ± 0.03	5.69 ± 0.02	5.72 ± 0.02
	40°C		5.63 ± 0.02	5.66 ± 0.02	5.65 ± 0.03	5.68 ± 0.02	5.68 ± 0.01	5.68 ± 0.02	5.70 ± 0.02

S4. Determination of Antibacterial Efficacy in Time at Different Storage Temperatures

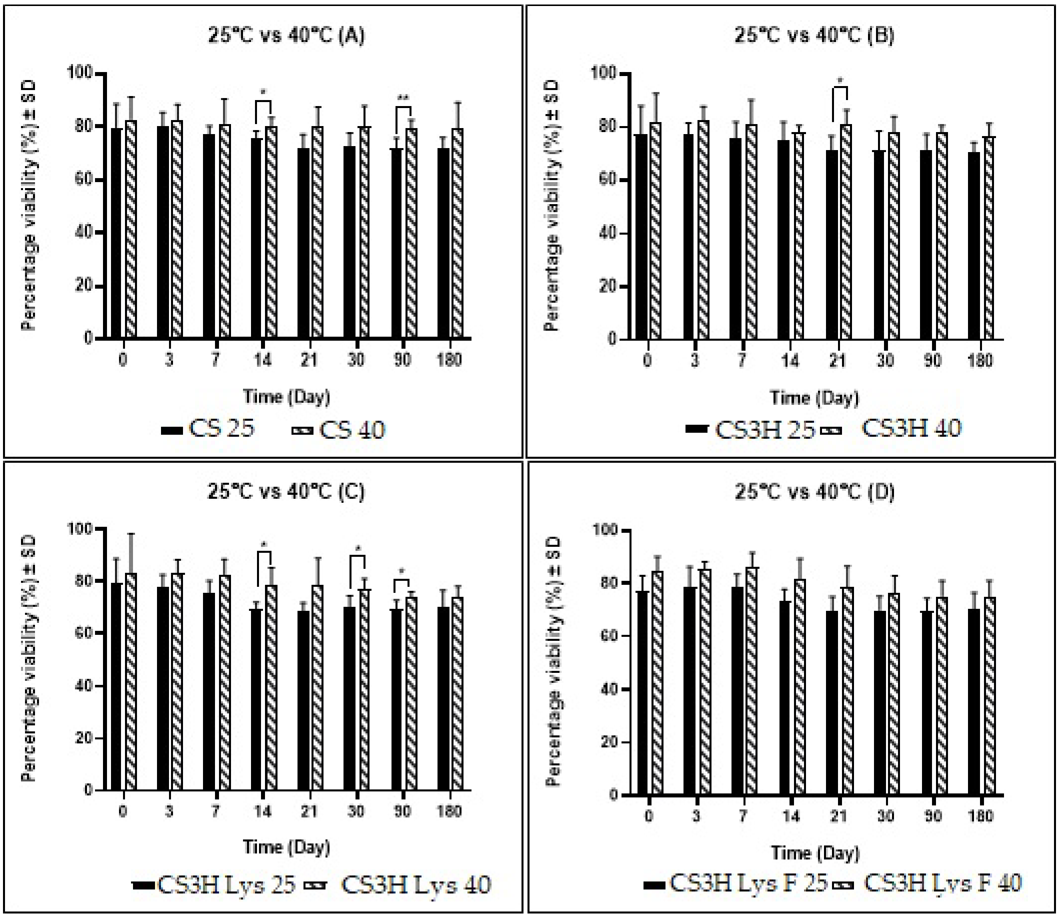

Figure S8. Effect of temperature on the percentage *S. mutans* biofilm formation in the presence of chitosan mouthwashes. A. CS, B. CS3H, C. CS3H Lys, and D. CS3H Lys F. Data are presented as mean ± SD (n = 9). Statistical significance of temperature at each time point was tested by two-way ANOVA and subsequently by Sidak's multiple comparison test. (* = $p \leq 0.05$, ** = $p \leq 0.01$).
